# Isolation of wheat thylakoids for protein analysis

**DOI:** 10.1016/j.mex.2023.102266

**Published:** 2023-06-20

**Authors:** Chao-Yan Chang, Jin-Ying Gou

**Affiliations:** a School of Life Sciences, Fudan University, Shanghai 200438, China; b College of Agronomy and Biotechnology, China Agricultural University, Beijing 100193, China

**Keywords:** Wheat, Chloroplast, Thylakoid, Western blot, Wheat thylakoids isolation

## Abstract

Thylakoids host a large number of proteins to confer photosynthesis and chemical biosynthesis essential for plant survival and growth. Successful isolation of high-quality thylakoids is the first step to studying the compositions and function of thylakoid protein and metabolites. Nevertheless, former studies isolated chloroplasts and thylakoids using a high-speed centrifuge with Percoll, which was expensive and unfriendly to the environment. The method presented here aims to establish a simple and inexpensive method to isolate high-quality thylakoids for protein analysis by utilizing sucrose instead of Percoll to reduce the cost and modify the centrifuge speed into the range usually used in labs.

## Graphical abstract

**Figure:**
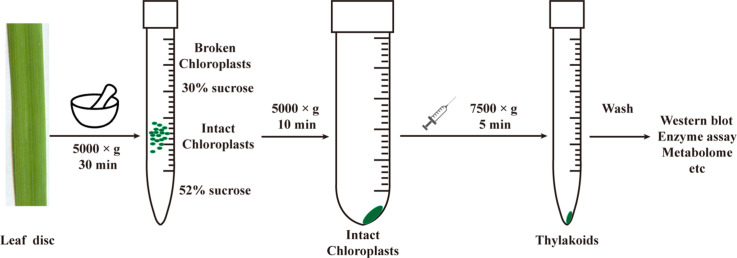

Specifications table

**Table:** 

Subject Area:	Agricultural and Biological Sciences
More specific subject area:	Protein and Proteomics
Protocol name:	Wheat thylakoids protein extraction
Reagents/tools:	D-sorbitol, KCl, EDTA disodium salt dihydrate, HEPES sodium salt, MgCl_2_·6H_2_O, Na_4_P_2_O_7_·10H_2_O, sucrose, NaH_2_PO_4_·2H_2_O, NaCl, sodium l-ascorbate, l-cysteine, and Bovine serum albumin (BSA).
Experimental design:	Extract chloroplasts from wheat leaves and isolate the intact ones between the 30% and 52% sucrose layer; break the chloroplasts using cold chloroplast shocking buffer and pellet thylakoids by centrifugation.
Method name:	Wheat thylakoids isolation
Trial registration:	Not applicable
Ethics:	Not applicable
Value of the Protocol:	Preparing High-quality thylakoids from wheat; In-expensive by using sucrose instead of Percoll; Uncomplicated and feasible by avoiding high-speed centrifuge.

## Description of protocol

### Methods details

#### Chemicals and reagents

BSA was purchased from Sigma-Aldrich Chemical Co. (St. Louis, USA) and kept in darkness at 4 °C. Tricine, D-sorbitol, KCl, EDTA disodium salt dihydrate, HEPES, sodium salt, MgCl_2_·6H_2_O, Na_4_P_2_O_7_·10H_2_O, sucrose, NaH_2_PO_4_·2H_2_O, NaCl, sodium l-ascorbate, and l-cysteine were purchased from the Shanghai Sangon Biochemical Co. (Shanghai, China). The plant-specific anti-actin mouse monoclonal antibody (AC009) was purchased from ABclonal (Shanghai, China). The anti-CP43 rabbit antibody (AS06–111) was purchased from Agrisera (Vännäs, Sweden). The pre-stained protein marker (WJ102) was purchased from Epizyme Biotech. Co. (Shanghai, China).

#### Sample treatment and protocol details

1. We spread wheat seeds in a petri dish on germination paper and rinsed them with tap water overnight. When the radicle length is equal to the seed length and the germ length reaches half of the seed length, it follows the standard for wheat seed germination. We selected healthy seeds with similar grain weight (about 45 mg) for germination and following analyses. Six germinated seeds were wrapped at the 1/3 from the top of a piece of germination paper (14.9 * 10.5 cm) and put in a glass cultivating pot filled with 3 cm depth tap water. The cultivating pot was kept in a growth chamber at 25 °C with 16 light/8 dark cycles for a week. Each sample consists of five healthy seedlings at the one-leaf/one-hart stage to minimize individual variations.
2. Prepare the chloroplast extraction buffer (Tricine, 20 mM, pH 8.4; D-Sorbitol, 300 mM; KCl, 10 mM; EDTA disodium salt dihydrate, 10 mM) and store it at 4 °C in the dark. Add BSA 0.25%(m/v), sodium l-ascorbate 4.5 mM, and l-Cystein 5 mM before use.
3. Fill the ice box about 3/4 full of ice, put a mortar on ice, and add 10 to 15 mL of chloroplast extraction buffer to keep it at a low temperature.
4. Cut the leaf discs of wheat into 1 cm lengths and grind them gently until the fibers are white. After the wheat leaves were fully ground, chloroplasts were released from the leaf tissues and resuspended in the extract buffer. Therefore, the remaining tissues remained as white fibers. Grind multiple times with only a few leaf discs, each time to maintain the chloroplast intact.
5. Filter the above samples with Miracloth (Merck-Millipore, Shanghai, China) into a clean 50 mL centrifuge tube. Drain out 1 mL and store it as the total protein.
6. Centrifuge at 1000 g for 10 min at 4 °C and drain out 1 mL of supernatant and store it as the soluble protein. Carefully drain out the residual supernatant and keep the pellets in ice until proceeding to the next step.
7. Resuspend the pellets with 4 mL of chloroplast extraction buffer and keep the sample in ice until proceeding to the next step.
8. Prepare the gradient buffer with 30% (m/v) and 52% (m/v) sucrose using the chloroplast extraction buffer.
9. Add 4 mL each of 52% (m/v) and 30% sucrose gently along the wall of a 15 mL clean centrifuge tube.
10. Carefully tap the tube to resuspend the pellets and remove the samples onto the top of the above gradient buffer.
11. Centrifuge at 5000 g for 30 min at 4 °C and collect the intact chloroplasts between the 30% and 52% sucrose layer into a clean 50 mL tube.
12. Add 20 mL of cold chloroplast washing buffer (HEPES, pH 7.8, 20 mM; D-Sorbitol, 300 mM; KCl, 10 mM; EDTA disodium salt dihydrate, 2.5 mM; and MgCl_2_·6H_2_O, 5 mM) and gently mix the samples.
13. Centrifuge at 5000 g for 10 min at 4 °C and drain the supernatants.
14. Repeat steps 12 to 13 for two more times.
15. Resuspend the pellets with 3 mL chloroplast washing buffer, keep the samples on ice, and store 300 µL of the sample as the chloroplast protein.
16. Add 20 mL of cold chloroplast shocking buffer (10 mM Na_4_P_2_O_7_, pH 7.8), gently mix the samples well, and keep them on ice for 30 min.
17. Pass the samples through a 1 mL syringe with a 24 G needle 50 times to break the chloroplast thoroughly.
18. Centrifuge at 7500 g for 5 min at 4 °C, drain out the supernatants and collect the pellets.
19. Repeat steps 17 to 18 one more time.
20. Resuspend the sample with 10 mL of thylakoid washing buffer I (Tricine, pH 7.8, 2 mM and sucrose, 300 mM).
21. Pass the samples through a 1 mL syringe with a 24 G needle 20 times, centrifuge at 4500 g for 5 min at 4 °C, and drain out the supernatants and collect the pellets.
22. Resuspend the sample with 10 mL of thylakoid washing buffer II (30 mM NaH_2_PO_4_, pH 7.8; MgCl_2_·6H_2_O, 5 mM; NaCl, 50 mM; sucrose, 100 mM; and EDTA disodium salt dihydrate, 1 mM).
23. Pass the samples through a 1 mL syringe with a 24 G needle 20 times and centrifuge at 4500 g for 5 min at 4 °C, drain the supernatants, and collect the pellets.
24. Resuspend the samples with 100 µL of thylakoid washing buffer II, normalize their contents based on the concentration of chlorophylls, separate them, and store them in a −80 °C freezer.

#### Method validation

We performed the above experiments and used antibodies recognizing target proteins in different cellular fractions [1] to validate the method. The normalized samples were mixed with 5 × SDS loading buffer, kept for 10 min at 65 °C, and separated in an SDS-PAGE. The proteins were transferred onto a PVDF membrane following a standard procedure [2]. Using polyclonal antibodies against actin (1:1000, ABclonal, Shanghai, China), CP43 (1:5000, Agrisera, Vännäs, Sweden), and ZEP1 (1:500, Abmart, Shanghai, China), their contents were analyzed in total, chloroplast, soluble, and thylakoid fractions [3]. According to the manufacturer's user manual, we used goat anti-rabbit mouse IgG HRP as the second antibody at 1:5000 dilution. We developed the samples with a Clarity™ Western ECL Substrate (Bio-Rad, USA) in Tanon 5200 Multi imaging system.

**Fig. 1:**
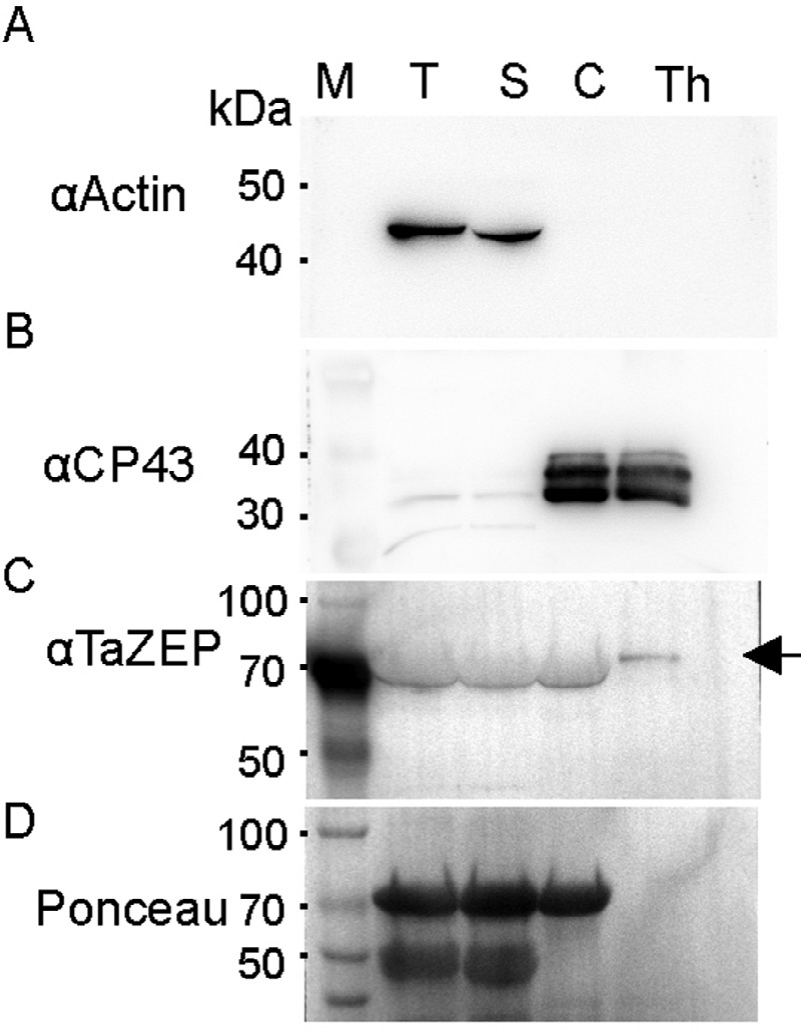
Western blot analysis of proteins resides in different cellular fractions. The antibodies dilution factors were 1:1000 (A), 1:5000 (B), and 1: 500 (C). The arrow in C points to the ZEP1 band. M, pre-stained protein molecular size marker. T, total protein. S, soluble protein. C, chloroplast. Th, thylakoids.

The monoclonal antibody against actin recognized a band in the total and soluble samples with a predicted molecular weight of 42 kDa, close to the expected actin molecular weight. There was no signal in the samples of chloroplast and thylakoids (Fig. 1A), suggesting no soluble protein contaminations. The antibody against CP43 (chloroplast protein 43) had strong signals in the chloroplast and thylakoid samples but not total or soluble samples (Fig. 1B). The above results agreed with the biological function of CP43 in the photosystem. We finally analyzed the above samples with a polyclonal antibody against wheat ZEP1 [4]. We detected a specific signal only in the thylakoid sample (Fig. 1C). The above data suggested that the samples prepared by this method were good enough for customer-made polyclonal antibodies. This work confirms the high importance of environmental sciences due to their various uses, as reported before [5], [6], [7], [8].

## Funding

This work was supported by the 10.13039/501100001809National Natural Science Foundation of China (31972350).

## CRediT authorship contribution statement

**Chao-Yan Chang:** Methodology, Data curation, Writing – original draft. **Jin-Ying Gou:** Conceptualization, Writing – review & editing.

## Declaration of Competing Interest

The authors declare that they have no known competing financial interests or personal relationships that could have appeared to influence the work reported in this paper.

## Data Availability

No data was used for the research described in the article.
